# Does higher cost mean better quality? evidence from highly-regarded adolescent drug treatment programs

**DOI:** 10.1186/1747-597X-2-23

**Published:** 2007-07-31

**Authors:** Bruce R Schackman, Erick G Rojas, Jeremy Gans, Mathea Falco, Robert B Millman

**Affiliations:** 1 Department of Public Health, Weill Cornell Medical College, 411 East 69^th ^Street New York, NY 10021, USA; 2 Drug Strategies, 1616 P Street, N.W. Suite 220 Washington, DC. 20036, USA

## Abstract

We conducted a survey to examine whether reimbursement levels are associated with the quality of adolescent substance use treatment programs in the United States. Between March and September 2005, telephone and written surveys were administered to program, clinical, and finance directors of previously surveyed highly regarded programs. Differences in quality scores were compared for programs with above versus below median reimbursement levels and examined in multivariate regression models constructed separately for programs offering residential and outpatient treatment. In residential treatment multivariate regression models, higher quality scores were associated with higher reimbursement, but this relationship was not observed for outpatient treatment. Even the highest level of outpatient reimbursement received may be too low to support quality improvement initiatives. Our results suggest that higher reimbursement may be a necessary component of quality improvement for residential adolescent drug treatment programs, and emphasize the need for further research to determine what levels of reimbursement and insurance coverage policies will encourage the expansion of high quality outpatient programs.

## Background

Although substance abuse is pervasive among American youth, only one in ten adolescents who need treatment actually receives help [1]. Adolescents differ from adults in terms of patterns of substance use, the influence of developmental and social factors, and the prevalence of co-occurring disorders [2]. Treatment experts agree that adolescent programs should not simply be adult programs modified for adolescents. Adolescent treatment programs must address the different contexts that shape an adolescent's life including school, recreation, peers, the distinctive nature of the juvenile justice system, and differences in medical care [3].

Costs are a major concern for parents and professionals who refer adolescents to treatment, particularly for residential programs. Charges for outpatient programs are generally lower, because most adolescents attend just two to five outpatient sessions per week, but they can still impost a financial burden. Private insurance spending for drug and alcohol abuse treatment has declined, while cost-sharing requirements have increased for those with insurance [4].

Substance abuse treatment providers report that lower reimbursement and greater administrative burdens are associated with difficulties attracting and retaining better-trained staff, and coping with overworked staff [5]. One would expect that higher reimbursement would also be associated with more treatment, more therapeutic hours or more frequent treatment contacts, which have been found to be associated with improved outcomes (e.g. fewer positive urinalysis results, fewer readmissions) [6,7]. Studies have evaluated the impact on substance abuse outcomes of reimbursement systems such as Medicaid managed care [8,9] and performance-based contracting [10,11], but we found no empirical studies that directly evaluate associations between reimbursement rates and substance abuse treatment program quality. However, high-quality pediatric and adolescent preventive care has been found to be associated with higher reimbursement [12], and higher quality child day care centers also have higher costs [13].

In a previous study, we evaluated the quality of 144 adolescent treatment programs identified as exemplary by professionals in the field, directors of state drug and alcohol agencies, and major national organizations [14]. To examine whether there is any direct association between the cost and quality of these programs, we collected new information on reimbursement levels in a follow-up survey.

## Methods

### Sample

In the previous study, alcohol and drug abuse agencies in all 50 states, several national organizations and federal agencies (including the American Medical Association, the American Academy of Pediatrics, and the National Institute of Drug Abuse), and members of an expert panel were requested to identify adolescent substance use treatment programs that they considered to be exemplary and thus highly regarded [14].

The sample for this study consists of 138 programs still in operation of the 144 programs that had been selected to participate in the previous study. Three surveys were conducted between March 2005 and September 2005: an in-depth telephone survey administered to program directors, and two telephone or written surveys administered to clinical and finance directors.

### Measures

Respondents to the finance director survey were asked to indicate the average reimbursement per day, per week or per stay by treatment setting and the proportion of reimbursements by type of payer (government, private insurer, self-pay). If residential reimbursement levels were reported in monthly or weekly units, these responses were divided by their respective time frame to have a uniform time unit for reimbursements. If reimbursement levels were reported per stay, these responses were divided by the average length of stay reported by the program.

In the previous study, a panel of 22 experts defined 9 key elements of effective treatment for adolescent substance abuse based on a review of the literature, interviews and discussions with panel members, and small working group recommendations. Open-ended survey responses were then coded according to 45 quality components based on these 9 key elements with a reliability of 0.79 as measured by Cronbach's α with standardized items [14]. In this study, the 45 quality components were condensed to a 20-item quality score according to the following procedure. First, patterns of missing data and the extent of variation in the responses were used to reduce the number of questions from 45 to 31. Then, a factor analysis was conducted on the remaining questions using a cut-off of 0.4 on factor loading [15]. The 20 questions with the highest factor loading explained 85% of the variation in the previous study survey responses. These 20 questions comprised the quality score for this study (see Appendix).

Additional variables that were repeated from the previous study included treatment approaches used (twelve-step, cognitive behavioral, therapeutic community, motivational enhancement, multi-systemic therapy, and multidimensional family therapy), geographical region, and accreditation. We also obtained data on other variables potentially associated with reimbursement level, including the presence of a waiting list to enter the program, referral sources, average daily client census, program treatment slots and average length of stay (inpatient) or treatment duration (outpatient). An occupancy ratio was calculated for each program that provided an average daily client census and program treatment slots.

### Analysis

Since reimbursement for residential services differs significantly from reimbursement for outpatient services, we conducted separate analyses for each of these services. The sample size was insufficient to develop three separate models for programs offering residential only, outpatient only, and both services. Differences in median quality scores were analyzed by reimbursement level and other program characteristics using the Mann-Whitney test, and we report approximate z-scores using this test. Because we consider this analysis to be exploratory in nature given the lack of relevant previous research conducted on this topic, all variables with p-values ≤ 0.25 were entered into linear regression models and final multivariate linear regression models were constructed using the backward elimination procedure [16]. An advantage of using this procedure over forward selection and stepwise regression is that the predictive capability of all variables of potential interest can be examined jointly [17].

## Results

Response rates were 87% for program directors, 60% for clinical directors, and 57% for financial directors. Table 1 provides an overview of program characteristics for all programs (n = 120) and for programs with sufficient data from all interviews to be included in regression models (n = 63). There are no notable differences between programs included versus excluded from the regression models, and approximately 40% of residential-only programs, 50% of outpatient-only programs, and 50% of programs providing both residential and outpatient services were included in the regression models. The mean daily reimbursement reported for residential services was $201 compared to $56 for outpatient services (Table 2). The mean quality score for all programs was 14.28 out of a possible 20 and for programs included in the regression models the mean quality was 14.77, with no significant differences by setting.

**Table 1:** Program characteristics

	**All Programs**		**Programs in Reduced Regression Models**			
			**Offering Residential Treatment**		**Offering Outpatient Treatment**	

	**N**	**%**	**N**	**%**	**N**	**%**

**Program Setting**						
Residential only	27	22.5	10	26.3	-	
Outpatient only	42	35.0	-		20	37.7
Multilevel	51	42.5	28	73.7	33	62.3
Program Approach*						
Twelve-step	94	80.3	32	84.2	40	75.5
Cognitive behavioral	105	89.7	36	94.7	47	88.7
Therapeutic Community	45	38.5	15	39.5	16	30.2
Motivational Enhancement	82	70.1	27	71.0	40	75.5
Multisystematic therapy	51	43.6	19	50.0	23	43.4
Multidimensional Family	61	52.1	18	47.4	23	43.4
**Region**						
South	36	30.0	7	18.4	12	22.6
Midwest	28	23.3	9	23.7	15	28.3
West	30	25.0	12	31.6	16	30.2
Northeast	26	21.7	10	26.3	10	18.9
Source of Referral*						
Hospital	46	38	17	44.7	23	43.4
Juvenile Justice System	107	89	32	84.2	49	92.4
Private Practitioner	58	48	19	50.0	28	52.8
State health agency	47	39	16	42.1	20	37.7
Adult Substance Abuse	17	14	6	15.8	9	17.0
Facility						
School System Official	83	69	22	57.9	42	79.3
Parent or Family Member	98	82	30	78.9	46	86.8
Insurance Company	34	28	13	34.2	17	32.1
Walk-ins	47	39	14	36.8	20	37.7
**Programs w/accreditation^†^**						
Yes	63	54.3	22	57.9	30	56.6
No	53	45.7	16	42.1	23	43.4
**Total Revenue >$1 million**	42	56.0	29	76.3	28	53.8
**Total Revenue <$1 million**	33	44.0	9	23.7	24	46.2
**Receive Grants**						
Yes	52	75.4	25	69.4	43	81.1
No	17	24.6	11	30.6	10	18.9
**Waiting List**						
Yes	37	50.7	26	70.3	26	49.1
No	36	49.3	11	29.7	27	50.9

Note: excludes missing responses.

* Multiple responses allowed

^† ^Joint Commission on Accreditation of Healthcare Organizations (JCAHO), Rehabilitation Accreditation Commission (CARF), Council on Accreditation (COA)

**Table 2:** Client utilization, reimbursement and quality score for residential and outpatient services

**Variable**	**N**	**Median**	**Mean**	**SD**
Programs offering residential treatment*				
Average daily client census	49	26.0	43.4	59.0
Client occupancy ratio (%)^†^	49	87.7	86.2	0.1
Average length of stay (days)	47	96.0	118.8	92.8
Daily residential reimbursement ($)	38	183.00	200.92	99.72
Quality Score^#^	78	15.0	14.6	1.6
Programs offering outpatient treatment*				
Average daily client census	59	20.0	39.7	57.1
Client occupancy ratio (%)^†^	47	50.0	55.9	0.3
Average treatment duration (days)	47	120.0	131.7	92.8
Daily outpatient reimbursement ($)	26	50.50	55.73	26.47
Quality Score^#^	93	15.0	14.3	1.9

* residential treatment = residential only or both residential and outpatient; outpatient treatment = outpatient only or both residential and outpatient

^† ^occupancy ratio = number of clients/number of available client slots

^# ^Maximum score is 20

Among programs offering residential services, factors individually associated with a higher quality score in bivariate analyses included higher average daily reimbursement (Mann-Whitney z = 2.12, p = 0.04), multisystemic therapy (Mann-Whitney z = 2.12, p = 0.03), motivational enhancement therapy (Mann-Whitney z = (-)2.05, p = 0.04), and accepting client referrals from hospitals (Mann-Whitney z = 2.22, p = 0.03), private practitioners (Mann-Whitney z = (-)2.92, p < 0.01), or insurance companies (Mann-Whitney z = 2.15, p = 0.03). However, the only significant predictor of a higher quality score in the final multivariate model for these programs was higher residential reimbursement (t = 2.24, df = 35, p = 0.03) (Table 3).

**Table 3:** Multivariate regression models predicting quality score among programs offering residential treatment

**A. Full Model**			**B. Reduced Model (backward elimination procedure)**		
R-square = 0.249, n = 38			R-square = 0.185, n = 38		

**Variable**	**β**	**p-value**	**Variable**	**β**	**p-value**

Residential Reimbursement Above Median	1.01	0.03	Residential Reimbursement Above Median	1.01	0.03
Residential Census Above Median	0.67	0.15	Residential Census Above Median	0.67	0.15
Motivational Enhancement Therapy	0.28	0.61			
Multisystemic Therapy	-0.03	0.96			
Receive Hospital Referrals	0.46	0.47			
Receive Private Practitioner Referrals	0.03	0.96			
Receive Insurance Company Referrals	0.41	0.49			
With Accreditation*	0.20	0.70			

* Joint Commission on Accreditation of Healthcare Organizations (JCAHO), Rehabilitation Accreditation Commission (CARF), Council on Accreditation (COA)

Among programs offering outpatient services, factors individually associated with a higher quality score in bivariate analyses included monthly staff training (Mann-Whitney z = 2.16, p = 0.03), having a waiting list (Mann-Whitney z = 2.22, p = 0.03), multisystemic therapy (Mann-Whitney z = 2.59, p = 0.01), and accepting client referrals from hospitals (Mann-Whitney z = 2.25, p = 0.02) and private practitioners (Mann-Whitney z = (-)2.43, p = 0.02). In the final multivariate model for these programs, a higher quality score was associated with having a waiting list to enter the program (t = 2.45, df = 48, p = 0.02), having received accreditation from a national organization (t = 3.07, df = 48, p < 0.01) and having received grants (t = 2.32, df = 48, p = 0.02); there was no significant association with reimbursement level (Table 4).

**Table 4:** Multivariate regression models predicting quality score among programs offering outpatient treatment

**A. Full Model**			**B. Reduced Model (backward elimination procedure)**		
R-square = 0.462, n = 23			R-square = 0.261, n = 53		

**Variable**	**β**	**p-value**	**Variable**	**β**	**p-value**

Outpatient Reimbursement Above Median	0.36	0.69	Waiting List	0.99	0.02
Waiting List	1.35	0.14	With Accreditation*	1.31	<0.01
With Accreditation*	1.62	0.14	Receive Grants	1.36	0.02
Receive Grants	1.20	0.37	Receive Insurance Company Referrals	0.85	0.08
Receive Insurance Company Referrals	1.48	0.21			
Motivational Enhancement Therapy	-0.49	0.68			
Multisystemic Therapy	0.74	0.31			
Receive Hospital Referrals	0.47	0.60			

*Joint Commission on Accreditation of Healthcare Organizations (JCAHO), Rehabilitation Accreditation Commission (CARF), Council on Accreditation (COA)

## Discussion

We conducted surveys of program, clinical, and finance directors of highly regarded adolescent substance use treatment programs to examine whether reimbursement levels are associated with program quality. Our analysis was subject to several limitations. Our surveys were limited to highly regarded programs and there was a relatively narrow range of variation in quality. Hence the effect size of reimbursement on quality was relatively small: an additional 1.0 quality point was associated with residential reimbursement above the median compared to a mean (standard deviation) quality score of 14.6 (1.6). With more complete data from programs with greater variations in quality and size, we could have seen a greater reimbursement effect.

Our cross-sectional design is vulnerable to reverse causality, in this instance the possibility that higher quality programs are able to obtain greater reimbursement due to consumer demand as opposed to higher reimbursement directly supporting better quality. However, most adolescent substance use treatment programs are non-profits and there is little evidence of high profit margins in this setting.

Our analysis indicates that higher cost residential adolescent programs deliver better quality treatment, suggesting that higher reimbursement may be a necessary component of quality improvement for these types of programs. However, we found no association between higher cost for outpatient programs and better quality treatment. High quality outpatient treatment appears to be recognized externally when it occurs, since better quality was associated with waiting lists, accreditation, and success in obtaining external funding. However, we believe that even the highest level of outpatient reimbursement received (e.g. $84 per day for the top quartile of outpatient reimbursement) is still too low to have an effect on quality. The great majority of adolescents receive treatment in outpatient settings rather than in residential settings [18]. Further research is needed to determine what levels of reimbursement and insurance coverage policies will encourage the expansion of high quality outpatient programs.

## Competing interests

The author(s) declare that they have no competing interests.

## Authors' contributions

BS, MF, and RM conceived of the study, devised the study design and participated in the completion of the manuscript. JG participated in the data collection and completion of the manuscript. ER participated in the statistical analysis and completion of the manuscript. MF and RM contributed expert opinion. All authors read and approved of the final manuscript.

## Appendix: 20-item Program Quality Score Questions

1. Does the program either provide mental health services for clients onsite or coordinate their care with community mental health providers?

2. Do you conduct some form of reassessment of clients during the course of treatment?

3. In screening and assessment process, does the program use either a standardized instrument* or a structured clinical interview?

4. In your screening and assessment process, does the program use a standardized mental health instrument*?

5. Does the treatment plan address mental health issues?

6. Do you provide the client's family with individual and/or multifamily therapy sessions?

7. Will you refer parents who are abusing substance to treatment?

8. Do you maintain contact with juvenile justice officials regarding clients who have been referred by the juvenile justice system?

9. Do you utilize a specific text or curriculum designed for adolescents?

10. Are adolescent clients typically treated only with other adolescents, as opposed to being integrated with adult clients?

11. Do you incorporate positive reinforcements, such as increasing responsibilities and/or privileges?

12. Do you utilize special recreational programming or offer courses of particular interest to adolescents?

13. Do you provide clients with gender-specific group sessions?

14. Does your program tailor itself for gay and lesbian youth?

15. Do you create a continuing care plan for the client beyond referring clients to outside services?

16. Does the program link clients with relevant community services upon discharge?

17. Do you collect any other information related to client outcomes after treatment?

18. Are there any evaluation or other types of studies completed on your program, and if so, may we receive a copy?

19. Do you have staff members with training in mental health issues?

20. Do all clinical supervisors possess at least a master's degree?

* Standardized instruments sources: [19,20]
